# Characterization of polycystic ovary syndrome among Flo app users around the world

**DOI:** 10.1186/s12958-021-00719-y

**Published:** 2021-03-03

**Authors:** Tarun Jain, Olivia Negris, Dannielle Brown, Isabel Galic, Rodion Salimgaraev, Liudmila Zhaunova

**Affiliations:** 1 Division of Reproductive Endocrinology and Infertility, Department of Obstetrics and Gynecology, Northwestern University Feinberg School of Medicine, 676 N. St. Clair, Suite #2310, Chicago, IL 60611-2914 USA; 2 Institute for Public Health and Medicine, Northwestern University Feinberg School of Medicine, Chicago, IL USA; 3 Flo Health, Inc., Wilmington, Delaware, USA

**Keywords:** Polycystic ovary syndrome, BMI, Bloating, Hirsutism, Menstrual cycle tracking

## Abstract

**Background:**

Polycystic ovary syndrome (PCOS) is a complex and multi-faceted endocrine disorder that affects 5–20% of women. Literature is limited regarding potentially differing PCOS phenotypes among women around the world.

**Objective:**

To use Flo app technology to understand the multifaceted characteristics of PCOS across several countries and identify contributing risk factors to the development of this condition.

**Study design:**

Flo is a widely used female health and wellbeing app with period tracking functionality that provides a globally representative and medically unbiased perspective on PCOS symptomatology. A chatbot dialog on PCOS was subsequently administered on the Flo application (app) to users from 142 countries (with at least 100 respondents) who have the app running in English during September–October 2019.

**Results:**

For analyses, we selected the five countries with the greatest number of respondents: US (*n* = 243,238), UK (*n* = 68,325), India (*n* = 40,092), Philippines (*n* = 35,131), and Australia (*n* = 29,926). Bloating was the most frequently reported symptom among PCOS-positive women and appeared to be the main predictor of PCOS in our model (odds ratio 3·76 [95% CI 3·60–3·94]; *p* < 0·0001). Additional top predictors of PCOS are high blood cholesterol and glucose levels. As BMI increased, the percentage of women who reported a physician-confirmed PCOS diagnosis also increased. However, women in India did not follow this trend.

**Conclusion:**

Our findings are based on the largest known PCOS dataset and indicate that symptoms are more complex than previously understood. The most frequently reported symptoms (bloating, facial hirsutism, irregular cycles, hyperpigmentation, and baldness) are broader than those included in the Rotterdam criteria. Future work should reevaluate and refine the criteria utilized in PCOS diagnosis.

**Supplementary Information:**

The online version contains supplementary material available at 10.1186/s12958-021-00719-y.

A. Why was the study conducted?
  To characterize PCOS and its varying symptomology around the world
B. What are the key findings?
  Among the five countries with the highest number of Flo app users, the highest ratio of PCOS positive users occurred in the Philippines, followed by India, UK, US, and Australia.
  Bloating was the most frequently reported symptom among PCOS-positive women and appeared to be the main predictor of PCOS in our model.
  As BMI increased, the percentage of women who reported a physician-confirmed PCOS diagnosis also increased.
C. What does this study add to what is already known?
  PCOS phenotypes are complex and vary significantly between countries.
  The most frequently reported symptoms (bloating, facial hirsutism, irregular cycles, hyperpigmentation, and baldness) are broader than those included in the Rotterdam criteria.

## Introduction

Polycystic ovary syndrome (PCOS) is an endocrine disorder affecting 5–20% of women worldwide. It is characterized by excess androgen production, ovulatory dysfunction, and menstrual irregularities. Individuals with PCOS are at increased risk for metabolic abnormalities and type 2 diabetes mellitus, infertility, obstetrical complications, obesity, endometrial carcinoma, and mood disorders [1]. Thus, PCOS presents a significant public health concern and requires comprehensive and consistent diagnostic criteria to accurately diagnose and care for the affected population [2].

Since the 1990 National Institutes of Health (NIH) PCOS conference, the syndrome has been recognized to have a spectrum of symptoms with some diagnostic criteria [3]. In 2003, the Rotterdam consensus refined criteria widely used today, which requires the presence of at least two of the following: hyperandrogenism, ovulatory dysfunction, and polycystic ovaries on ultrasound [4, 5]. In contrast, the Androgen Excess Society (AES) supports that excess androgen is a critical feature of PCOS and defines the condition as the presence of hyperandrogenism and ovarian dysfunction [6]. The use of varying PCOS diagnostic criteria raises issues of compatibility for PCOS research worldwide, resulting in confusion within clinical practice and a “delay in progress in understanding the syndrome” [7]. While ethnic variation of PCOS is becoming better understood, discrepancies exist and research on geographical location and environmental exposure in relation to PCOS symptomatology warrants further investigation.

Symptoms of hyperandrogenism include hirsutism, acne, androgenic alopecia, and virilization [8]. Hirsutism is present in up to 80% of patients with hyperandrogenism and thus is the most commonly used clinical diagnostic criterion [8]. Acne affects roughly 15–25% of patients with PCOS and varies with ethnicity [9]. Moreover, ovulatory dysfunction typically refers to menstrual cycle irregularities and subfertility [1]. One retrospective study followed 786 women with PCOS and found 66% reported infertility [10]. Furthermore, PCOS is closely associated with obesity as 30–75% of PCOS patients are obese [11, 12]. Women who are obese are more likely to have severe hyperandrogenism, menstrual disturbances, and psychological disturbances in PCOS [12–17]. Due to this worsened disease manifestation, overweight and obese women may be more likely to receive a PCOS diagnosis [18]. Despite the known association between PCOS and obesity, the prevalence of obesity in women with PCOS differs by age, ethnicity, and geographical location [19, 20]. Research into variability of PCOS with regard to body mass index (BMI) is limited by the lack of population-based studies.

While it is established that PCOS may vary in clinical presentation, literature is limited regarding differing phenotypes across a globally representative sample. There is a need to better understand geographic variation in PCOS, and also to improve women’s satisfaction with treatment of PCOS. A 2017 study found that over one-third of women spent over two years, visiting at least three medical providers, searching for a diagnosis [21]. Additionally, only 25% of these women were satisfied with the recommendations they received [21]. The inconsistency in diagnoses may be due to the lack of data on the presentation of PCOS from a medically unbiased sample. Previous literature has shown that the participant referral population has significant effects on the PCOS phenotype presented due to factors such as patient concern and access to care [22].

The use of innovative health and wellbeing apps such as Flo, provides an opportunity to estimate the characteristics of PCOS in a globally representative and medically unbiased population. Due to the geographically dispersed use of Flo, we are able to analyze PCOS symptomology relative to country in order to better characterize PCOS and its differing phenotypes among users worldwide. This present study aims to use Flo app technology to understand the multifaceted characteristics of PCOS across several countries and identify contributing risk factors to the development of this condition.

## Methods

### Flo application (app) description and functionality

Flo (https://flo.health) is the most downloaded AI-driven women’s health tracking app worldwide (according to AppAnnie, 2020, iOS, by downloads) with over 150 million users. Flo encompasses over 36 million monthly active users, with the majority (55%) located in the US and Europe. Flo is currently available in over 200 countries in 22 languages on iOS and Android.

**Fig. 1:**
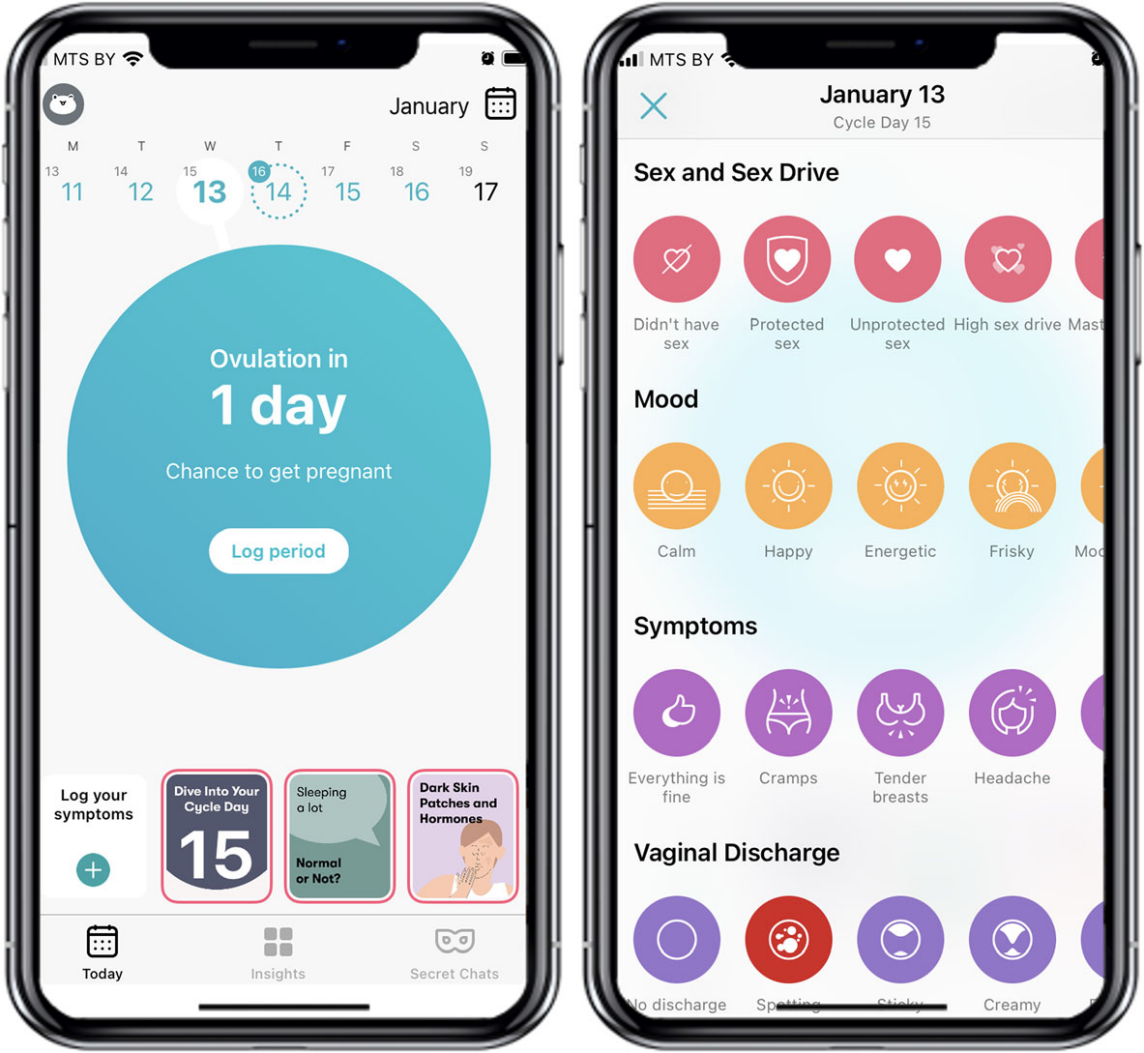
Sample screenshots of the Flo app. On the left, the app displays what day the user is currently on in their cycle. On the right, a user can choose symptoms in ‘Sex and Sex Drive’, ‘Mood’, ‘Symptoms’, ‘Vaginal Discharge’ categories

The Flo app is a health and wellbeing platform that supports women during their entire reproductive lives – from first menstruation to early motherhood and menopause. Flo also allows a user to track symptom information on the symptom panel screen that consists of 84 symptoms, and contains categories like sex and sex drive, mood, symptoms (cramps, tender breast, headache etc), vaginal discharge, physical activity, menstrual products and other. Flo users can also enter their ovulation and pregnancy test results as well and the use of contraceptive, water intake, sleep hours, and weight (Fig. 1).

Flo relies on AI algorithms to make personalized period and ovulation predictions. In addition, Flo applies AI algorithms to provide its users with personalized, evidence-based, and expert-reviewed courses and articles on women’s health and well-being. Flo also provides its users with a secured place to discuss intimate topics, ask questions anonymously, and get support from millions of women worldwide. Finally, Flo functionality includes various chatbots that proactively initiates a user dialogue on various health topics, give data feedback and real time health alerts based on tracked symptoms.

Flo is a home-friendly app and is not obligatory during doctor interviews. Nevertheless, Flo users who are using the app for at least six months have access to a Health Report that summarizes average period and cycle length for the past six months as well as symptoms and signs logged by a user each cycle. A Health Report can also be downloaded and shared with the doctor (see Supplementary File 1).

For more information, Flo can also be downloaded for free from Apple App store or Android’s Google Play.

### Data collection

Data related to PCOS symptoms and past diagnoses were obtained from the PCOS chatbot dialog available to Flo users during September–October 2019. The dialogue included 18 questions related to reproductive and general health (a detailed list of questions and answers used in the chatbot is available from the authors upon request). The PCOS dialog was released in English and was available to 5.4 million Flo users who have their app running in English. Both iOS and Android users participated in the questionnaire.

**Fig. 2:**
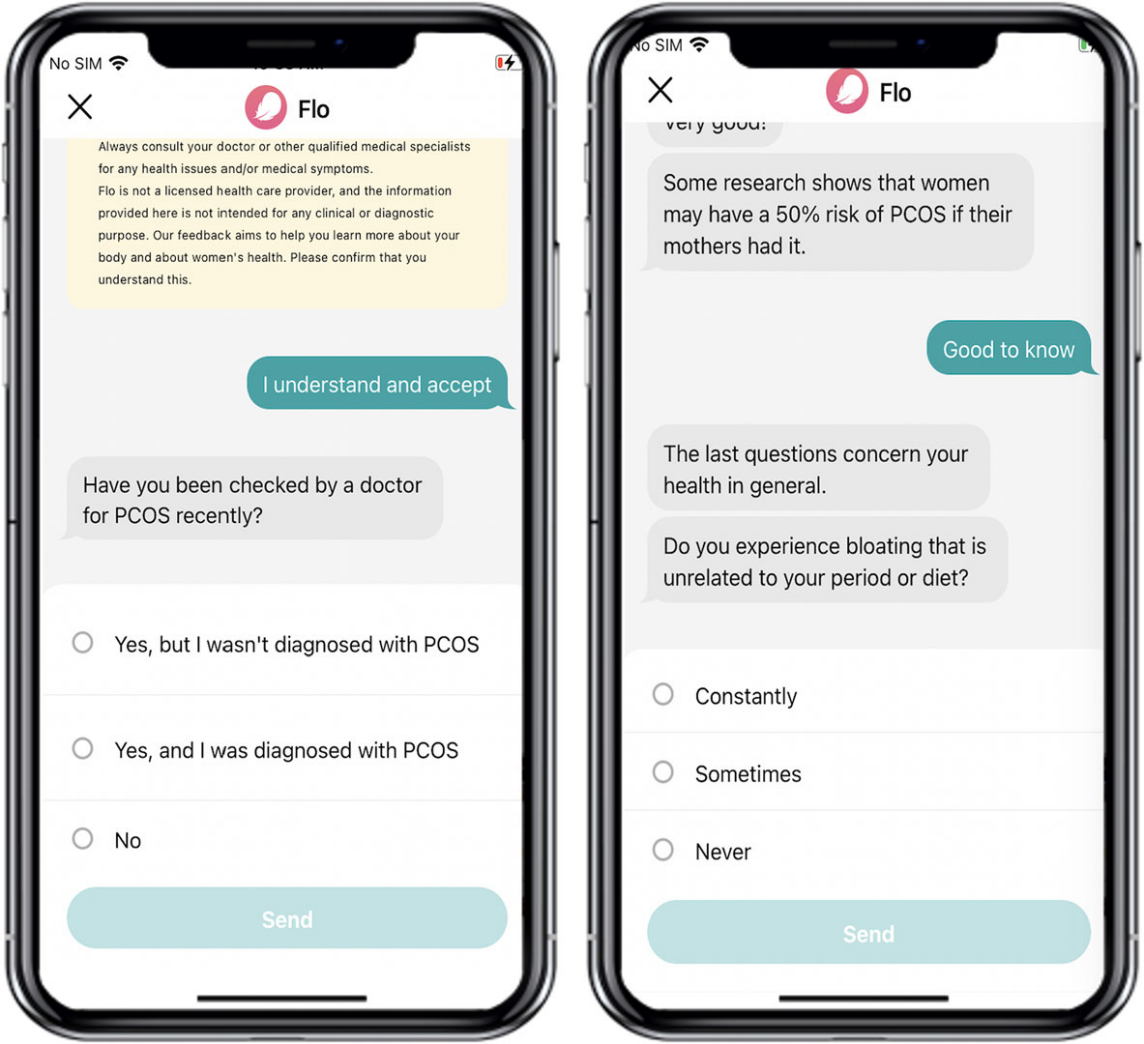
Sample screenshots of the Flo app with examples of questions asked to the user in the process of the dialog with options for answers

The set of questions and answers for users were the same for all countries. PCOS diagnosis status was defined based on the user’s responses to the question “Have you been checked by a doctor for PCOS recently?”. Only users with a definite diagnosis were included in the study analysis such as users who responded “Yes, and I was diagnosed with PCOS” or “Yes, but I wasn’t diagnosed with PCOS”). Users who replied “No” were not considered in this study and excluded from analysis (Fig. 2).

The PCOS chatbot also inquired questions about symptoms that a user experienced: e.g. “Have you experienced persistent acne (for more than 6 months) that usual skincare doesn’t clear up?” with “Yes” or “No” answers.

Study inclusion criteria included women aged 18–44 years seeking to track their cycle or to conceive, who were not pregnant or after a pregnancy, and who were not on active contraception. Participant characteristics including age and BMI were also collected from Flo users during the sign-up process. All users in the study agreed to the use of their de-identified and aggregated data for research purposes. Institutional review board (IRB) approval for this study was obtained from Northwestern University.

### Data preparation

A user dataset from the PCOS chatbot dialog was used to analyze the effect of PCOS symptoms as predictors of disease. A second dataset based on the results of the same chatbot dialog, which also included user BMI information, was used to analyze the impact of BMI on the odds ratio of having PCOS. Further analyses were conducted using only the data of women coming from the top five countries with the highest number of survey respondents.

### Statistical analysis

Binary logistic regression was used as a statistical method. The diagnosis of PCOS was used as a dependent variable in the regression model. The dependent variable could have one of two values: ‘PCOS positive’ or ‘PCOS negative’ (the participants with an undefined diagnosis were not included in the model). Separate self-reported symptoms of the disease, with two possible levels of the variable -- presence or absence of a symptom, were used as predictors to determine the odds ratio of PCOS and to estimate 95% confidence intervals (CI). A separate logistic model was created for individual predictors for each country and the total (of the top five countries) group.

To estimate the statistical significance of the difference in average weight between PCOS positive and PCOS negative groups, a two-sided t-test was used. To analyze the influence of BMI as a predictor of PCOS, participants were divided into six BMI groups with normal BMI (18.5–24.9) used as a reference. BMI was used as a dichotomous variable that can take one of two values: normal BMI and a comparable group (for example, 18.5–24.9 vs. 25.0–29.9) with subsequent relative odds ratio and 95% CI assessment. Separate models were created for each of the BMI groups for each country and the total (of the top five countries) group. Statistical methods were implemented in Statsmodels and Scipy libraries of Python 3.

## Results

**Fig. 3:**
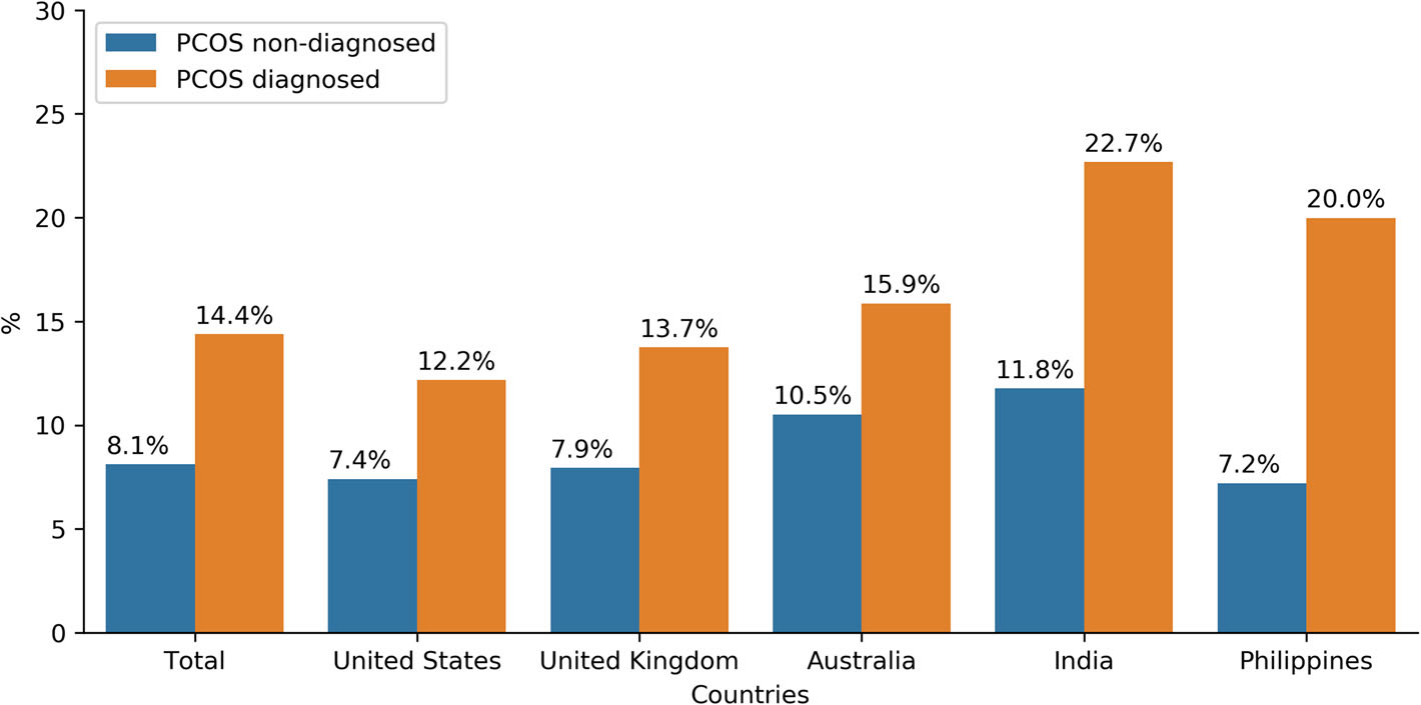
Percentage of women with physician-confirmed PCOS versus women without PCOS among users from top five responding countries

The cohort includes responses from Flo users in 142 countries with at least 100 respondents in total. Most English-speaking Flo users who started the PCOS chatbot dialog were located in the US (*n* = 243,238), followed by the UK (*n* = 68,325), India (*n* = 40,092), Philippines (*n* = 35,131), and Australia (*n* = 29,926); these countries are referred to as the top five based upon number of responding users. Of the top five cohort, 14.4% self-reported having PCOS that was previously diagnosed by a physician. 8.1% of users self-reported a negative PCOS status and 77.5% had not recently been checked by a physician, indicating that their PCOS status is unknown. Across all the users from the top five countries, the ratio of PCOS positive users to PCOS negative users was 1.8 (see Table 1). Among the top five countries, the highest ratio of PCOS positive users to PCOS negative users occurred in the Philippines (2.8), followed by India (1.9), UK (1.7), US (1.7), and Australia (1.5). Countries with at least 1000 respondents with a confirmed PCOS diagnosis, but not included in the top five analyses with the highest ratios of PCOS positive to PCOS negative users were: Trinidad and Tobago (3.0), United Arab Emirates (2.2), Jamaica (1.8), New Zealand (1.5), and Pakistan (1.5) (see Table 2). As shown in Fig. 3, a physician’s diagnosis of PCOS was most frequently reported in India (22.7%) followed by the Philippines (20.0%).

**Table 1:** Demographic and PCOS self-reported diagnosis status among responding Flo app users

	Mean Age ± SD	PCOS Diagnosed (n, %)	PCOS not Diagnosed (n, %)	PCOS Status Unknown (n, %)	Ratio PCOS (+) / PCOS (−)
Overall (top 5 countries)	27.2 ± 5.82	59,871 (14.37)	33,824 (8.11)	323,017 (77.52)	**1.77**
United States	27.3 ± 5.97	29,619 (12.18)	18,002 (7.4)	195,617 (80.42)	**1.65**
United Kingdom	29.7 ± 5.97	9387 (13.73)	5421 (7.93)	53,517 (78.33)	**1.73**
India	25.02 ± 4.71	9096 (22.69)	4721 (11.78)	26,275 (65.54)	**1.93**
Australia	27.28 ± 5.77	4747 (15.86)	3149 (10.52)	22,030 (73.61)	**1.51**
Philippines	24.7 ± 5.45	7022 (19.99)	2531 (7.2)	25,578 (72.81)	**2.77**

**Table 2:** Countries with at least 1000 PCOS negative and PCOS positive responding users altogether, ordered by PCOS (+) / PCOS (−) ratio

	PCOS Not Diagnosed	PCOS Diagnosed	Total	Ratio PCOS (+) / PCOS (−)
**Trinidad and Tobago**	369	1110	1479	3.01
**Philippines**	2531	7022	9553	2.77
**United Arab Emirates**	569	1225	1794	2.15
**India**	4721	9096	13,817	1.93
**Jamaica**	433	764	1197	1.76
**United Kingdom**	5421	9387	14,808	1.73
**United States**	18,002	29,619	47,621	1.65
**Australia**	3149	4747	7896	1.51
**New Zealand**	516	763	1279	1.48
**Pakistan**	1109	1617	2726	1.46
**Canada**	2457	3361	5818	1.37
**Malaysia**	606	765	1371	1.26
**Indonesia**	719	866	1585	1.2
**South Africa**	987	1154	2141	1.17
**Ireland**	501	572	1073	1.14
**Nigeria**	743	661	1404	0.89
**Romania**	624	440	1064	0.71

In the US, UK, and Australia, the most common symptoms of PCOS positive women were bloating, facial hirsutism, and irregular cycles (see Table 3). In the Philippines, women with PCOS were most likely to have bloating (75.4%), hyperpigmentation (68.5%), irregular cycle (64.4%), and baldness (61.0%). Women with PCOS in India most often reported baldness (74.4%), hyperpigmentation (66.5%), and irregular cycles (62.2%). In four of the top five countries, the most frequently reported symptom was bloating, seen in 73.8% of US women, 78.6% of UK women, 80.4% of women in Australia, and 75.4% of women in the Philippines with PCOS.

**Table 3:**
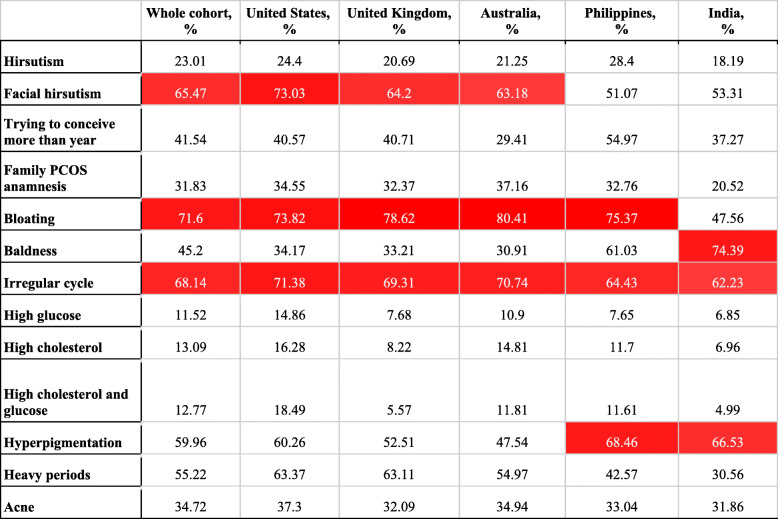
Prevalence of symptoms among women with PCOS from the top 5 responding countries

Key: The top 3 most prevalent symptoms for each country are highlighted in red

As indicated in Table 4, we examined the associations for predictors of self-reported PCOS according to symptom manifestation. Within the whole cohort of the top five countries, the most prevalent predictors of PCOS were bloating (OR: 3.8; 95% CI: 3.6–3.9; *p* < 0.0001), both high cholesterol and glucose (OR: 3.6; 95% CI: 3.3–3.9; *p* < 0·0001), and high glucose alone (OR: 2.9; 95% CI: 2.7–3.1; *p* < 0.0001). Bloating was one of the most common predictors of self-reported PCOS among users in all countries with the exception of India. Likewise, having both high cholesterol and glucose was a strong predictor of self-reported PCOS among users in all countries with the exception of the Philippines. Users in India most commonly reported high cholesterol and glucose (OR: 2.8; 95% CI: 2.1–3.8; *p* < 0·0001) followed by an irregular cycle (OR:2.8; 95% CI: 2.4–3.2; *p* < 0·0001) and high glucose (OR: 2.6; 95% CI: 2.0–3.3; *p* < 0·0001). In the US and Philippines, facial hirsutism was also a strong predictor of self-reported PCOS (OR: 1.2 and 3.6, respectively).

**Table 4:**
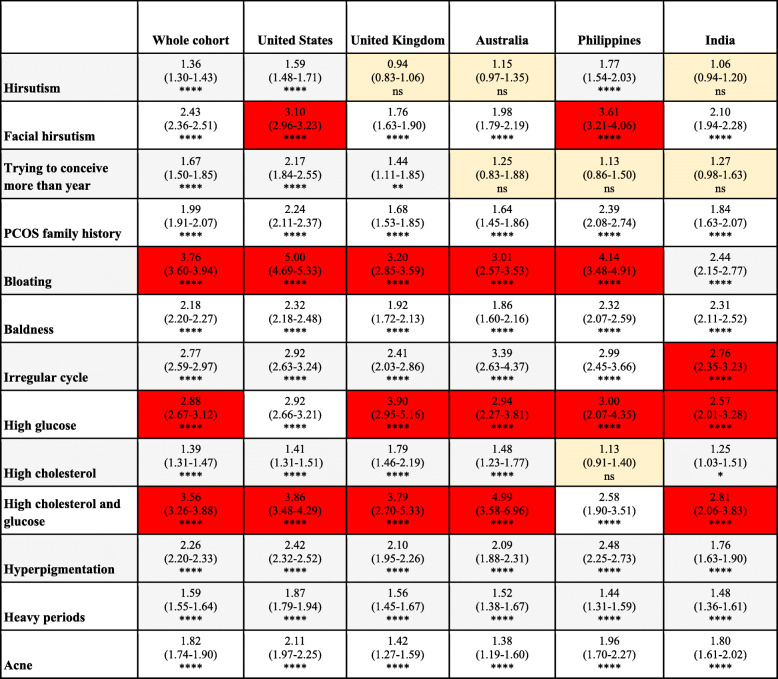
Odds of having PCOS among women with self-reported symptoms compared to symptomless women

Data presented as: Odd ratio (95% CI)

ns = *P* > 0.05, * = *P* ≤ 0.05, ** = *P* ≤ 0.01, *** = *P* ≤ 0.001, **** = *P* ≤ 0.0001

Key: The top 3 highest odds of self-reported symptoms for each country are highlighted in red, while non-significant odds are highlighted in yellow

**Fig. 4:**
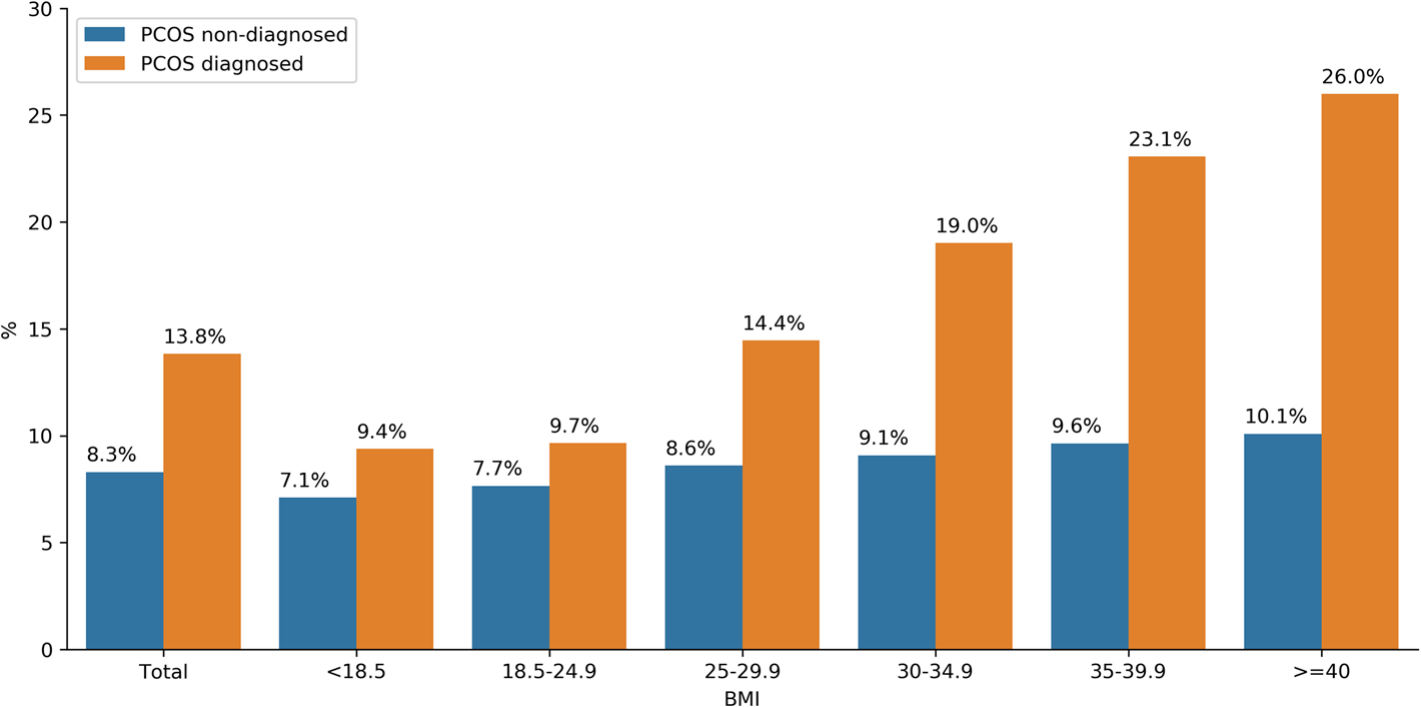
Percentage of women with physician-confirmed PCOS versus women without PCOS stratified by BMI from top five responding countries

There was a statistically significant difference between overall mean BMI for PCOS positive users in the top five countries (27.7) and the overall mean BMI for PCOS negative users in the top five countries (26.2) (*p* < 0·0001). Across all BMI groups, there is an observed trend that as BMI increased, the percentage of women with a self-reported PCOS diagnosis also increased (see Fig. 4). While this percentage of women with PCOS increases, the percentage of women without a PCOS diagnosis stays relatively stable across BMI groups.

Compared to women of normal weight, women who are overweight, obese, severely obese, and morbidly obese all had a significantly increased odds of having a positive PCOS diagnosis in the top five cohort (see Table 5). There is again a positive trend relating this increased risk of presenting with PCOS and increasing BMI across the whole cohort.

**Table 5:**
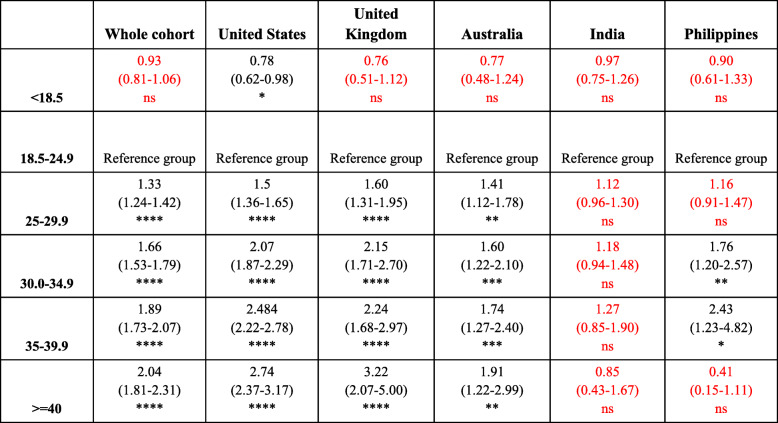
Odds of having PCOS between different BMI groups compared to women with normal BMI (reference group)

Data presented as: Odd ratio (95% CI)

ns = *P* > 0.05 (red), * = *P* ≤ 0.05, ** = *P* ≤ 0.01, *** = *P* ≤ 0.001, **** = *P* ≤ 0.0001

Key: The non-significant odds are highlighted in red

In the US and UK, women who are obese and severely obese were at least twice as likely to have a positive PCOS diagnosis compared to women of normal weight (US: Obese OR: 2.1, Severely Obese OR: 2.5; UK: Obese OR: 2.2, Severely Obese OR = 2.2; *p* < 0.05). A similar, but less pronounced, relationship exists for obese and severely obese women in Australia and the Philippines (Australia: Obese OR: 1.6, Severely Obese OR: 1.7; Philippines: Obese OR:1.8, Severely Obese OR: 2.4; *p* < 0.05). The greatest risk exists for morbidly obese women in the UK as they had an over three times increase in odds of having a PCOS diagnosis compared to normal weight women (OR: 3.2; p < 0.05). Interestingly, no significant relationship between BMI and odds of a PCOS diagnosis exist for women in India (Table 5).

## Discussion

Using Flo’s technology, we analyzed the largest known PCOS symptom dataset to obtain a comprehensive understanding of the distribution of PCOS and its varying phenotypes worldwide. Among all countries, the highest ratio of PCOS positive to PCOS negative users occurred in Trinidad and Tobago, Philippines, United Arab Emirates, India, Jamaica, UK, followed by the US. The US, UK, India, Philippines and Australia had the greatest number of respondents to the PCOS dialog box. Within these top five countries, the most prevalent predictors of PCOS were bloating, both high cholesterol and glucose, and high glucose alone. Additionally, among four of the top five countries, bloating was the most frequently reported symptom. When examining BMI in relation to PCOS, there is a trend that as BMI increased, the percentage of women with a self-reported PCOS diagnosis also increased. However, women in India did not follow this trend as there was no significant relationship between BMI and PCOS status.

Previous research on global PCOS symptomatology is both limited and inconsistent. Many have identified South Asian women to have among the lowest prevalence rates, yet this group has been found to have high rates of insulin resistance and metabolic syndrome [23–26]. Another study found that 52% of women residing in India present with PCOS, which is the highest reported prevalence internationally [24]. Consistent with this report, our findings show that India and the Philippines were among the top countries with high ratios of PCOS positive to PCOS negative users. Another study analyzing PCOS phenotypes among different populations reported that women with PCOS from Asia and America were at increased risk of type II diabetes [17]. Their PCOS is most often characterized by insulin resistance, high BMI, or central obesity while Europeans and Middle Eastern women often experience androgenic alopecia, hirsutism, and hyperandrogenism [17, 27]. In our sample from India, individuals who reported both high cholesterol and high glucose were almost three times more likely to self-report having PCOS. However, respondents in the US and UK with symptoms of both high cholesterol and high glucose were almost four times more likely, and those in Australia almost five times more likely to report PCOS. Moreover, East Asian women with PCOS often have a milder hyperandrogenic phenotype and lower BMI compared to others, but have the highest prevalence of metabolic syndrome [23]. Kumarapeli et al. studied a semi urban population in Sri Lanka and found that of women with self-reported oligo/amenorrhea or hirsutism, over 90% had a confirmed PCOS diagnosis [28]. These women tend to have less hirsutism compared to women from Europe and the US.

It is known that the prevalence of PCOS is increased in overweight and obese women, and that obesity prevalence has globally increased in the last few decades [29–31]. Our results also reveal that as BMI increased, the proportion of women with a PCOS diagnosis also increased. However, obesity prevalence is highly variable by age, ethnicity, and geographical location [19, 20]. In the US and UK, obese women were twice as likely to have PCOS compared to those of normal weight; while there were no observed trends in BMI and odds of PCOS diagnosis in India. Geographic differences in the prevalence of obesity is likely a result of the interaction between individual factors (e.g. genetic) and environmental factors (e.g. food supply) [32]. A previous meta-analysis indicated that an increased risk of obesity exists for Caucasian women from the US and Europe compared to Asian women from China and Taiwan, suggesting a difference in the nature of PCOS based on location [18]. Understanding such geographical differences in PCOS as it relates to BMI is critical for countries where increased obesity exists, as overweight and obese PCOS patients are more likely to exhibit clinical signs of androgen excess, significantly more severe insulin resistance, as well as anxiety and depression [33–38].

Compared to the NIH diagnostic criteria, the more expansive definition and inclusion of additional phenotypes of the Rotterdam and AES criteria may explain the greater estimates of PCOS prevalence [39]. When using the same defining criteria, variations in the reported prevalence across countries can in part be explained by ethnic differences, by the approaches used to define study population(s), and the application of varying methods to evaluate key PCOS features [40]. In surveying the largest known sample on identified PCOS symptoms, we are able to provide evidence that the symptomatology may be more complex than previously understood. Within the top five countries, our most frequently reported symptoms were bloating, facial hirsutism, irregular cycles, hyperpigmentation, and baldness. The symptoms reported in our sample are broader than those included in the Rotterdam criteria, suggesting more work and further research is needed to reevaluate and refine PCOS diagnostic criteria. Also, the most frequently reported symptoms of PCOS varied across countries, suggesting the presence of environmental and/or genetic effects on the PCOS phenotype. We find it unique and interesting that although women in India had the most frequently reported diagnosis of PCOS (22.7%), these women exhibited a different phenotype relative to women from the other countries. Women in India with PCOS were significantly less likely to experience bloating relative to the other countries examined. Furthermore, women in India with PCOS uniquely did not exhibit a significant relationship between BMI and PCOS status. Possible reasons for this unique phenotype may include differences in genetics, diet, and environmental exposures. Further research is needed to confirm and better understand these differences. Interestingly, we found that symptoms were similar between US/UK and between India/Philippines - countries that are socio-demographically similar.

Gynecological and reproductive education delivered through apps has potential to improve physician to patient interactions, while also providing large quantities of menstrual cycle and related data [41, 42]. There are over a hundred female health and wellbeing apps with more than 200 million downloads [43]. As such, medical professionals and researchers can gather information from large, unselected patient populations like ours in order to improve the understanding of gynecological disorders such as PCOS. Flo and other fertility apps can also provide public health benefits by offering standardized health promotion messages during various stages of reproductive life [42].

Strengths of our study included a very large global sample of medically unbiased women. A limitation is that women who already have certain medical conditions may have been more likely to participate in the dialog. In addition to the fact data were self-reported, women who said they did not a have physician-confirmed PCOS diagnosis may have another reproductive disorder which might be symptomatically similar to PCOS. It is also possible that different countries use different diagnostic criteria and medical professionals may have different approaches to PCOS diagnosis. Lastly, the dialog was available to Flo users running the app in English, which limited representation especially in countries that are not predominantly English speaking.

## Conclusion

Via analysis of a worldwide PCOS dataset, we obtained a more comprehensive understanding of the distribution of PCOS and its varying phenotypes. The most frequently reported symptoms were bloating, facial hirsutism, irregular cycles, hyperpigmentation, and baldness, which are broader than those included in the Rotterdam criteria. Future work should reevaluate and consider refining criteria utilized in diagnosing and caring for the many women with PCOS around the world.

## Supplementary Information

**Additional file 1.**

## Data Availability

The data that support the findings of this study are available on request from the corresponding author, Dr. Tarun Jain. The data are not publicly available due to the privacy of information provided by Flo App user participants.

## Abbreviations

PCOS: Polycystic ovary syndrome
app: Flo application
NIH: National Institutes of Health
AES: Androgen Excess Society
IRB: Institutional review board
BMI: Body mass index
CI: Confidence intervals
