# Supplementary material for: Characterization of polycystic ovary syndrome among Flo app users around the world
Source: Reprod Biol Endocrinol. 2021 Mar 3;19:36. doi: 10.1186/s12958-021-00719-y (PMC7927251; doi:10.1186/s12958-021-00719-y)
Supplement: Supplementary file 1 — Additional file 1. [file 12958_2021_719_MOESM1_ESM.pdf]

HEALTH REPORT Made by Flo

www.flo.health

COVERAGE: **30 June 2020 - 30 December 2020**

EXPORTED: **7 December 2020**

The cycle report made by Flo is based on at least the last 6 cycles you logged in the app. To get more accurate cycle predictions and more reliable health insights, please log more information about your health condition.

Cycle and Period Length

AVERAGE CYCLE LENGTH: **30 days**

AVERAGE PERIOD LENGTH: **6 days**

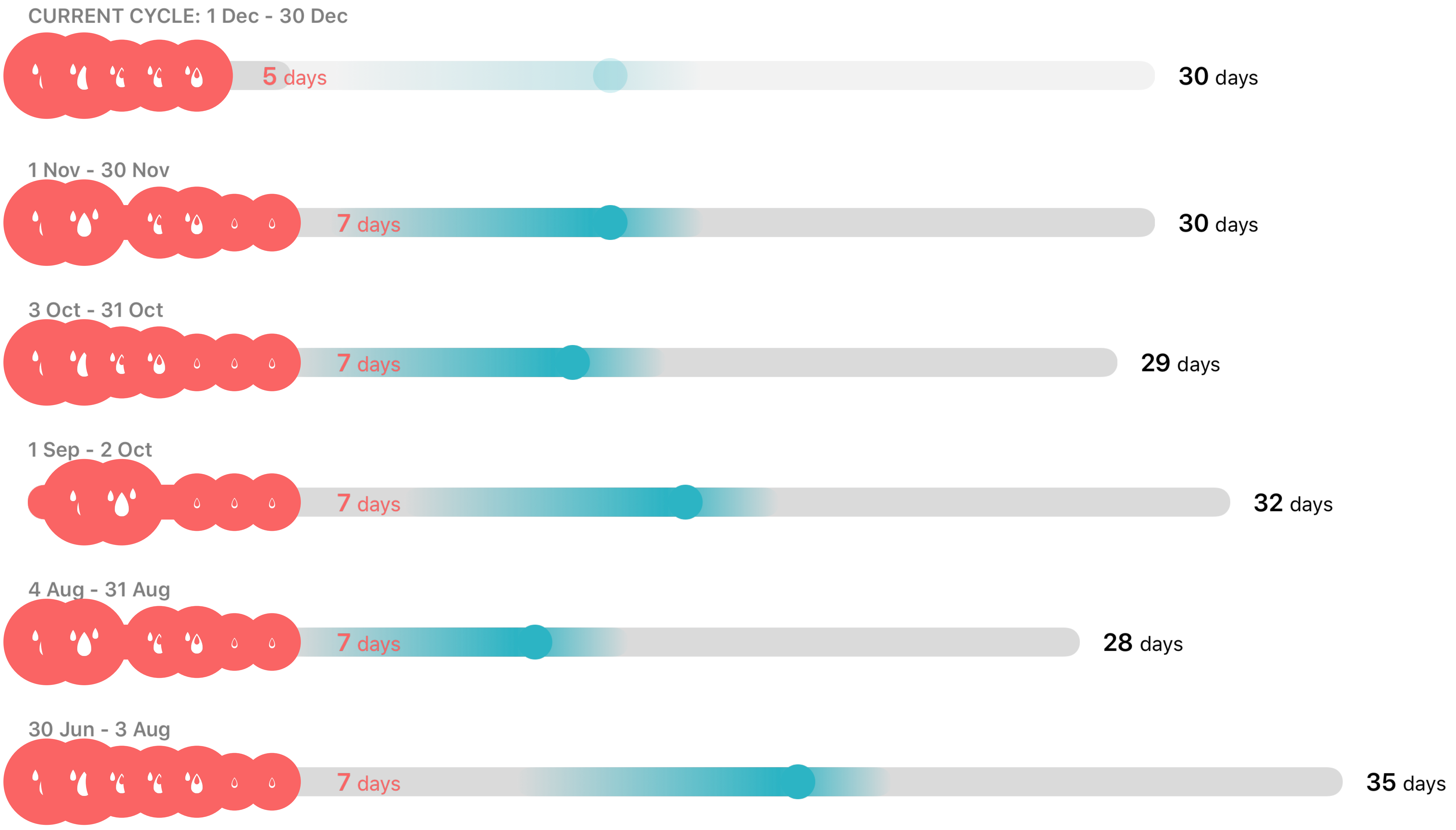

Your Symptoms at a Glance

1. It was common for you to log **cramps** on day 1-5 of your cycle.
2. You constantly logged **bloating** 15-17 days before your cycle ended.
3. **Headache** was typical for you on day 1-3 of your cycle.
4. You regularly logged **headache** 14-18 days before your cycle ended.

5 It was common for you to log **headache** 1-3 days before your cycle ended.

6 **Backache** was typical for you on day 1 of your cycle.

7 1-3 days before your cycle ended, you repeatedly logged **backache**.

8 You regularly logged **cravings** 15-17 days before your cycle ended.

9 **Obsessive thoughts** was typical for you on day 1-2 of your cycle.

10 **Diarrhea** was typical for you on day 1-4 of your cycle.

11 You regularly logged **insomnia** on day 1-5 of your cycle.

12 **Happy** usually happened 14-16 days before your cycle ended.

13 It was common for you to log **frisky** 15 days before your cycle ended.

14 15-18 days before your cycle ended, you repeatedly logged **energetic**.

15 **Sex** usually happened 14-17 days before your cycle ended.

16 It was common for you to log **acne** 1-3 days before your cycle ended.

17 1-3 days before your cycle ended, you repeatedly logged **tender breasts**.

18 14-18 days before your cycle ended, you often logged **eggwhite**.

HEALTH REPORT Made by Flo

www.flo.health

Your average cycle with commonly logged events

The cycle report made by Flo is based on at least the last 6 cycles you logged in the app. To get more accurate cycle predictions and more reliable health insights, please log more information about your health condition.

(1) The number of times the event was logged on a specific day during the last 6 cycles.

TOP 5 MOST LOGGED SYMPTOMS

CYCLE DAY	1	2	3	4	5	6	7	8	9	10	11	12	13	14	15	16	17	18	19	20	21	22	23	24	25	26	27	28	29	30	31	32	33	34	35
Headache	4	2	1												2			1										1		1		1		1	1
Cramps	4	5	2		1																														
Acne	4	2			1																							1		1		1		1	1
Tender breasts	3	2																										1		1		1		1	1
Backache	4																											1		1		1		1	

DISCHARGE

CYCLE DAY	1	2	3	4	5	6	7	8	9	10	11	12	13	14	15	16	17	18	19	20	21	22	23	24	25	26	27	28	29	30	31	32	33	34	35
Eggwhite	1												1	1	3		1			1	1														
Creamy					1									1	1																			1	
Sticky							1							1																					
Watery		1													1																				
No discharge						1	1																												
Unusual																													1						
Spotting																														1					

MOOD

CYCLE DAY	1	2	3	4	5	6	7	8	9	10	11	12	13	14	15	16	17	18	19	20	21	22	23	24	25	26	27	28	29	30	31	32	33	34	35
Calm		1	2	2		3	3																												
Energetic			1		1	1	1							1		1	1			1															
Obsessive thoughts	2	2																											1						
Frisky				1											1			1			1														
Anxious	1	1																														1			
Apathetic	2	1																																	
Happy													1	1							1														
Very self-critical	2																																		
Depressed		1																											1						
Feeling guilty																												1	1						
Confused	1																																		
Sad																														1					
Irritated		1																																	

SEX

CYCLE DAY	1	2	3	4	5	6	7	8	9	10	11	12	13	14	15	16	17	18	19	20	21	22	23	24	25	26	27	28	29	30	31	32	33	34	35
Protected sex				1			1								1	1	1	1			1									1					
Unprotected sex															1	1	1	1																	
